# 单中心晚期非小细胞肺癌免疫治疗真实世界数据分析

**DOI:** 10.3779/j.issn.1009-3419.2019.11.02

**Published:** 2019-11-20

**Authors:** 艳霞 刘, 同梅 张, 远 高, 阳 曲, 葆华 鲁, 红梅 张, 群慧 王, 杰 李, 范彬 胡, 宝兰 李

**Affiliations:** 101149 北京，首都医科大学附属北京市胸科医院，北京市结核病胸部肿瘤研究所综合科 Department of General Medicine, Beijing Chest Hospital, Capital Medical University/Beijing Tuberculosis and Thoracic Tumor Research Institute, Beijing 101149, China

**Keywords:** 肺肿瘤, 免疫治疗, 临床疗效, 不良反应, Lung neoplasms, Immunotherapy, Clinical efficacy, Adverse events

## Abstract

**背景与目的:**

近些年，多项临床试验显示免疫检查点抑制剂（immunocheckpoint inhibitor, ICI）为晚期非小细胞肺癌（non-small cell lung cancer, NSCLC）患者带来生存获益，但临床试验有着严格而复杂的纳入与排除标准，其结果不能完全反映真实世界的实际情况。本研究拟探讨真实世界中免疫治疗的临床疗效和安全性以及可能相关的预后因素。

**方法:**

回顾性分析2017年1月-2019年7月在北京胸科医院接受免疫治疗的晚期NSCLC患者，收集患者基本临床资料、治疗疗效、无进展生存期（progression-free survival, PFS）和药物不良反应等资料，探讨临床疗效、不良反应及可能相关的预后因素。

**结果:**

研究共纳入34例患者，中位PFS为5.66个月（95%CI：4.48个月-6.84个月），1级-2级不良反应和3级-4级不良反应发生率分别为61.71%（22/34）和14.71%（5/34），共有3例（8.82%）患者出现致死性免疫相关不良反应（immune-related adverse event, irAE），其中2例为免疫相关肺炎，1例为免疫相关心肌炎。单因素分析显示肿瘤-淋巴结-转移（tumor-node-metastasis, TNM）分期、转移部位与中位PFS相关（*P* < 0.05），多因素分析显示存在肺外转移（OR=6.42, *P*=0.029）、胸膜转移（OR=14.14, *P*=0.006）为患者PFS的独立预后因素。

**结论:**

真实世界中免疫治疗对晚期NSCLC患者具有良好的疗效，但其严重irAE的发生率也较高。存在肺外转移、胸膜转移是接受免疫治疗的晚期NSCLC患者的不良预后因素。

## Abstract (en)

**Background and Objective:**

In recent years, a number of clinical trials have shown that immunocheckpoint inhibitors (ICI) have brought survival benefits to patients with advanced non-small cell lung cancer (NSCLC), however, such clinical trials comprise cohorts selected based on strict and complex entry and exclusion criteria, and the

**Results:**

cannot fully reflect the real world situation. The purpose of this study was to investigate the clinical efficacy and safety of immunotherapy in the real world, as well as possible prognostic factors.

**Methods:**

Patients with advanced NSCLC receiving immunotherapy in Beijing Chest Hospital from January 2017 to July 2019 were retrospectively collected, and the following information were collected: curative effect, progression-free surival (PFS) and adverse reactions. The occurrence of adverse reactions and clinical curative effect and prognosis factors that may be relevant were explored.

**Results:**

34 patients were enrolled in this study, median PFS was 5.66 months (95%CI: 4.48-6.84), grade 1-2 and 3-4 incidence of adverse events was 61.71% (22/34) and 14.71% (5/34), there were 3 patients (8.82%) experienced fatal immune related adverse events (irAE), 2 cases were immune associated pneumonia, 1 case was immune related myocarditis. Univariate analysis showed that tumor-node-metastasis (TNM) stage and metastatic site were correlated with median PFS (*P* < 0.05), and multivariate analysis showed that patients with extrapulmonary metastasis (OR=6.42, *P*=0.029) and pleural metastasis (OR=14.14, *P*=0.006) had shorter median PFS. Conclusion In the real world, immunotherapy has good efficacy in patients with advanced NSCLC, but the incidence of severe irAE is also higher. Distant metastasis and pleural metastasis are poor prognostic factors for advanced NSCLC patients receiving immunotherapy.

以免疫检查点抑制剂（immunocheckpoint inhibitor, ICI）为代表的免疫治疗极大地改变了晚期非小细胞肺癌（non-small cell lung cancer, NSCLC）的治疗模式。临床试验中ICI单药治疗二线及以上NSCLC患者的5年生存率达15%及以上^[1, 2]^，极大地改善了晚期NSCLC患者的预后，已成为一线化疗失败晚期NSCLC的标准治疗方案。在初治患者中，基于KEYNOTE-024试验^[3]^，ICI单药获批用于无驱动基因突变/融合细胞程式死亡-配体1（programmed cell death 1 ligand 1, PD-L1）表达≥50%的晚期NSCLC的一线治疗，而对于PD-L1表达 < 50%的无驱动基因突变/融合晚期NSCLC，Keynote-189^[4]^、Impower-150^[5]^等试验结果表明ICI联合化疗较化疗更能为患者带来生存获益。

免疫治疗正在迅速地改变着晚期NSCLC的治疗策略，虽然疗效可观，总体副反应发生率低，但是其特有的免疫相关副反应一旦出现，发展迅速，若不及时发现和治疗，将会加速疾病的发展，甚至危及生命^[6]^。然而，由于临床试验均基于特定标准选择的患者队列，因此，其结果不能完全外推至真实世界的患者，真实世界中使用ICI患者的临床病理特征、临床疗效、预后及副反应与临床试验中是否一致均需要真实世界的数据来回答。本研究回顾性地分析了2017年1月-2019年7月在首都医科大学附属北京胸科医院接受免疫治疗的34例晚期NSCLC患者的病例资料，总结临床病理特点、诊治情况、疗效、预后及不良反应，并初步分析预后相关因素和副反应发生情况，旨在探讨ICI在真实世界中治疗晚期NSCLC的临床疗效和安全性，以及与疗效和副反应相关的可能因素。

## 1 资料与方法

### 1.1 临床资料收集

回顾性收集2017年1月-2019年7月在首都医科大学附属北京胸科医院接受免疫治疗的晚期NSCLC患者，采集患者的年龄、性别、病理类型、吸烟史、驱动基因变异[表皮生长因子受体（epidermal growth factor receptor, EGFR）/鼠Kirsten肉瘤病毒致癌基因同源物（v-Ki-ras2 Kirsten ratsarcoma viral oncogene homolog, KRAS）/间变性淋巴瘤激酶（anaplastic lymphoma kinase, ALK）/*ROS*原癌基因l受体酪氨酸激酶（ROS proto-oncogene 1 receptor tyrosine kinase, ROS1）/鼠类肉瘤病毒癌基因同源物B1（v-raf murine sarcoma viral oncogene homolog B1, BRAF）]、PD-L1表达、Karnofsky体力状况（Karnofsky performance status, KPS）评分、肿瘤-淋巴结-转移（tumor-node-metastasis, TNM）分期、转移部位等基本临床特征以及治疗方法、临床疗效、预后及副反应。

### 1.2 纳入与排除标准

（1）纳入标准：美国东部肿瘤协作组（Eastern Cooperative Oncology Group, ECOG）PS评分0分-2分；重要脏器功能正常；细胞学或组织学确诊的非小细胞肺癌；按照第8版国际肺癌TNM分期标准进行分期的Ⅲb期-Ⅳ期患者。曾接受ICI治疗的患者。（2）排除标准：无明确病理诊断信息；同时患有其他肿瘤者或者5年内曾患其他肿瘤者；PS≥3分者；合并重要脏器功能不全者；需要长期服用皮质类激素治疗者；有系统性免疫疾病患者；有严重精神障碍者；数据收集时未完成1次疗效评价的患者。

### 1.3 疗效评定和不良反应

按照实体瘤疗效评价标准（Response Evaluation Criteria in Solid Tumors, RECIST）1.1版将近期疗效分为完全缓解（complete response, CR）、部分缓解（partial response, PR）、疾病稳定（stable disease, SD）和疾病进展（progressive disease, PD）。客观缓解率（objective response rate, ORR）=（CR+PR）/（CR+PR+SD+PD）×100%；疾病控制率（disease control rate, DCR）=（CR+PR+SD）/（CR+PR+SD+PD）×100%；无进展生存期（progression-free surival, PFS）定义为自免疫治疗开始之日至随访至患者病情进展或者死亡的时间，失访者及未进展者按照截尾值处理，截尾时间为确认其未进展的末次随访时间。不良事件根据美国国立癌症研究院通用毒性标准（National Cancer Institute Common Terminology Criteria for Adverse Events, NCI-CTCAE）4.03版进行判定，分为Ⅰ级-Ⅴ级。本研究中将Ⅲ级-Ⅳ级定义为中重度不良反应，Ⅴ级为死亡。

### 1.4 PD-L1表达和基因突变的检测

肿瘤组织中PD-L1表达水平的检测采用免疫组化方法，使用22C3 pharmDx的PD-L1抗体（Dako, USA）。肿瘤驱动基因变异（*EGFR*/*KRAS*/*ALK*/*ROS1*/*BRAF*）的检测采用ARMS荧光定量PCR法，使用艾德人类*EGFR*/*ALK*/*ROS1*基因突变联合检测试剂盒（中国）和艾德人类*KNBP*基因突变检测试剂盒（中国）。

### 1.5 随访

采用查阅病例、电话和复诊等方式进行随访，随访截止日期为2019年7月20日。

### 1.6 统计学分析

采用SPSS 23.0和GraphPad Prism 7软件进行统计分析和作图。以*Kaplan*-*Meier*检验估计中位PFS，采用*Log*-*rank*检验进行组间比较。采用*Cox*回归模型进行PFS的多变量分析。以*P* < 0.05为差异具有统计学意义。

## 2 结果

### 2.1 患者基本资料

根据纳入与排除标准，共纳入34例患者，其中位年龄为60.5岁；基因变异情况：纳入1例EGFR基因21外显子L858R突变的患者。基线资料如性别、病理类型、吸烟史、PD-L1表达、PS评分、TNM分期、转移部位等详见表 1。

**1:** 接受免疫治疗的34例晚期非小细胞肺癌患者的临床特征 Clinical characteristics of 34 patients with advanced non-small cell lung cancer receiving immunotherapy

Clinical features	*n*	Percentage
Gender		
Male	27	79.41%
Female	7	20.59%
Histology		
Adenocarcinoma	19	55.88%
Squamous	14	41.18%
Adenocarcinoma+Squamous	1	2.94%
Smoking history		
Yes	24	70.59%
No	10	29.41%
PD-L1 expression levels		
< 50%	17	50.00%
≥50%	9	26.47%
Unknown	8	23.53%
EGFR and ALK		
Wild-type (both)	24	70.59%
Mutant	1	2.94%
Unknown	9	26.47%
PS score		
0	7	20.59%
1	27	79.41%
TNM stage		
Ⅲ	6	17.65%
Ⅳa	17	50.00%
Ⅳb	11	32.35%
Metastasis status		
No metastasis	6	17.65%
Pulmonary metastasis	14	41.17%
Pleural metastasis	7	20.59%
Distant metastasis	7	20.59%
PS: performance status; TNM: tumor-node-metastasis; PD-L1: programmed cell death 1 ligand 1; EGFR: epidermal growth factor receptor; ALK: anaplastic lymphoma kinase.		

### 2.2 治疗方案与治疗周期

34例患者全部接受免疫检查点抑制剂治疗，其中7例接受BGA-A317治疗，14例接受纳武单抗（Nivolumab）治疗，2例接受派姆单抗（Pembrolizumab）治疗，8例接受信迪利单抗治疗，3例接受阿特朱单抗（Atezolizumab）治疗；所有患者均接受了至少2个周期及以上的治疗，中位治疗周期为7个周期（2个-20个周期）。以ICI为一线治疗的8例，其中2例接受ICI单药治疗，6例接受ICI联合化疗，其中位治疗周期为8个周期（4个-20个周期）；以ICI为二线治疗的21例，其中20例接受ICI单药治疗，1例接受ICI联合抗血管药物，其中位治疗周期为7个周期（2个-20个周期）；以ICI为三线及以上治疗的5例，其中3例接受ICI单药治疗，1例接受ICI联合化疗及靶向药物，1例接受ICI联合手术及放疗，其中位治疗周期为5个周期（2个-10个周期）。各治疗组的PD-L1表达情况和基因变异情况详见表 2。

**2:** 各治疗组的PD-L1表达情况以及基因变异情况 PD-L1 expression levels and gene mutation status in each treatment group

	First-line	Second-line	Subsequent to second-line
PD-L1 expression levels			
< 50%	3	12	2
≥50%	2	6	1
Unknown	3	3	2
*EGFR* mutation			
Positive	0	0	1
Negative	7	13	4
Unknown	1	8	0
*KRAS* mutation			
Positive	0	0	0
Negative	6	10	5
Unknown	2	11	0
*ALK* mutation			
Positive	0	0	0
Negative	6	15	4
Unknown	2	6	1
*ROS1* mutation			
Positive	0	0	0
Negative	5	9	2
Unknown	3	12	3
*BRAF* mutation			
Positive	0	0	0
Negative	5	6	3
Unknown	3	15	2

### 2.3 治疗疗效

34例患者的ORR和DCR分别为17.65%（6/34）和76.47%（26/34）。其中以免疫治疗为一线、二线、三线及以上治疗的ORR分别为50.00%（4/8）、9.52%（2/21）和0.00%（0/5），DCR分别为100.00%（8/8）、66.67%（14/21）和80.00%（4/5）。各治疗组的疗效评价见图 1。

**1:**
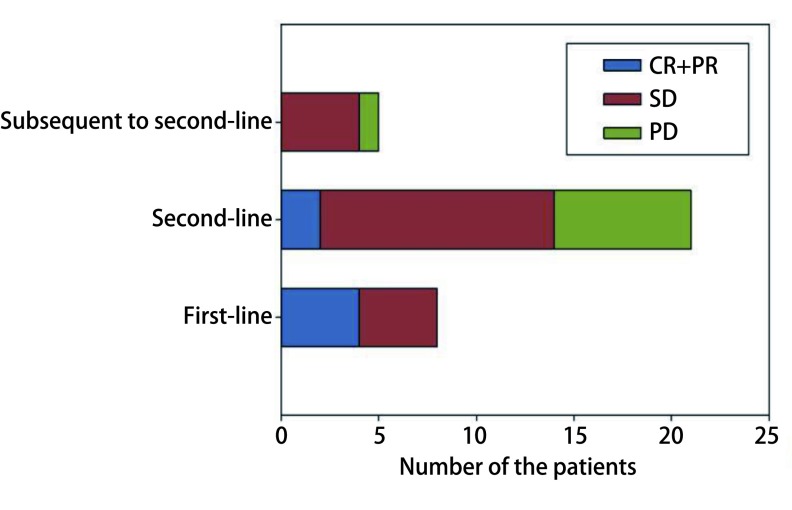
各治疗组的疗效评价 Efficacy evaluation of each treatment groups. CR: complete response; PR: partial response; SD: stable disease; PD: progressive disease.

### 2.4 不良反应

34例患者的总体不良反应的发生率为61.71%（22/34），1级-2级不良反应和3级-4级不良反应发生率分别为61.71%（22/34）和14.71%（5/34）。3例（3/34, 8.82%）患者出现4级免疫相关不良反应并最终致使患者死亡，其中2例为免疫相关肺炎，1例为免疫相关心肌炎。具体不良反应见表 3。

**3:** 不良反应的发生情况 Treatment-related adverse events according to category and grade

Adverse events	Grade Ⅰ	Grade Ⅱ	Grade Ⅲ	Grade Ⅳ	Percentage (all grades)
Glutamic oxaloacetic transaminase/Alanine aminotransferase elevation	7	2	1	0	29.41%
Hypothyroidism	5	4	0	0	26.47%
Skin rash	5	2	0	0	20.59%
Pneumonitis	0	1	1	2	11.76%
Renal dysfunction	3	0	0	0	8.82%
Digestive dysfunction	1	1	0	0	5.88%
Hyperglycemia	2	0	0	0	5.88%
Myocarditis	0	0	0	1	2.84%
Adrenal insufficiency	0	1	0	0	2.84%
Blurred vision	1	0	0	0	2.84%
Hyperglycemia	2	0	0	0	5.88%

### 2.5 预后

截至末次随访时间2019年7月20日，34例患者的中位随访时间为9.27个月。28例患者免疫治疗后疾病进展，6例患者接受免疫治疗中，病情平稳。11例患者死亡，1年生存率为61.64%。34例患者中位PFS为5.66个月（95%CI: 4.48-6.84）。一线、二线、三线及以上免疫治疗组的中位PFS分别为6.25个月（95%CI: 4.45-8.05）、5.78个月（95%CI: 4.04-7.48）和3.97个月（95%CI: 2.24-5.07）。

*Kaplan*-*Meier*单因素分析显示TNM分期、转移部位与患者PFS相关（*P* < 0.05），见图 2与表 4。

**2:**
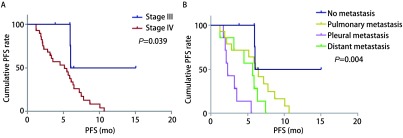
生存曲线。A：不同TNM分期的无进展生存曲线；B：不同转移部位的无进展生存曲线。 Survival curves. A: The PFS curves with different TNM stage; B: The PFS curves with different metastatic site.

**4:** 患者临床特征与中位无进展生存期相关性的单因素分析 Univariate analysis of correlation between clinical characteristics and median progression-free survival (PFS)

Clinical features	*n*	Median PFS (mo)	95%CI	*P*
Age (yr)				0.725
≤60	22	5.54	3.93-7.15	
﹥60	12	5.87	4.23-7.51	
Gender				0.993
Male	27	5.78	4.38-7.18	
Female	7	5.14	3.12-7.16	
Smoking history				0.363
Yes	24	6.12	4.61-7.63	
No	10	4.56	2.96-6.15	
Histology				0.071
Adenocarcinoma	11	7.00	3.66-5.79	
Squamous	19	4.72	4.63-9.35	
PD-L1 expression levels				0.268
< 50%	17	4.91	3.62-6.21	
≥50%	9	7.98	4.12-11.84	
Lymph-node metastasis				0.489
Yes	27	5.43	4.10-6.76	
No	7	6.59	4.17-9.01	
TNM stage				0.039
Ⅲ	6	10.50	6.06-16.94	
Ⅳ	28	4.97	3.91-6.02	
Metastasis present				0.004
No metastasis	6	10.50	6.06-16.94	
Pulmonary metastasis	14	5.83	4.14-7.52	
Pleural metastasis	7	3.05	1.85-4.25	
Distant metastasis	7	4.87	3.30-6.44	
PS score				0.487
0	7	6.30	3.86-8.74	
1	27	5.48	4.11-6.85	
Immune-related adverse event				0.723
Yes	22	5.13	4.48-7.27	
No	12	5.87	3.27-6.96	
Skin rash				0.056
Yes	7	7.86	5.07-10.64	
No	27	4.93	3.87-5.99	
Hypothyroidism				0.751
Yes	9	5.68	4.07-7.30	
No	25	5.76	4.15-7.36	

将单因素分析中*P* < 0.2以及临床上认为与预后相关的因素进行*Cox*多因素分析显示存在肺外转移（OR=6.42, *P*=0.029）、胸膜转移（OR=14.14, *P*=0.006）的患者中位PFS更短，见表 5。

**5:** 患者临床特征与中位PFS相关性的多因素分析 Multivariate analysis of correlation between clinical characteristics and median PFS

Factor	OR	95%CI	*P*
Pulmonary metastasis	3.199	0.603-16.968	0.172
Distant metastasis	6.419	1.210-34.039	0.029
Pleural metastasis	14.142	2.139-63.518	0.006
PD-L1 expression≥50%	0.777	0.260-2.318	0.650
Skin rash	0.341	0.116-1.002	0.050

## 3 讨论

近些年来，免疫治疗的不断发展持续更新着晚期NSCLC的治疗策略和理念。与化疗和小分子酪氨酸激酶抑制剂（tyrosine kinase inhibitor, TKI）不同，ICI通过恢复和增加细胞毒性T细胞（cytotoxic T cell, CTL）的免疫活性，逆转肿瘤微环境的免疫抑制状态，增强内源性抗肿瘤免疫效应，一旦发生应答，可能产生持久的临床反应^[2]^，理论上有治愈肿瘤的潜能，但临床实践表明，只有少数患者能从免疫治疗中长期获益。随着精准医学的发展，免疫治疗在晚期NSCLC患者中虽疗效可观，但缺乏有效的疗效和副反应预测的标志物，与预期的精准治疗有一定的差距。真实世界中如何有效、精准使用ICI仍需要临床医生不断探索，回顾分析真实世界数据能为我们带来新的启示。

受限于药物获批和适应证等原因，本研究纳入的ICI治疗患者也包含部分ICI临床研究入组的患者，治疗药物以PD-1抑制剂为主（31/34, 91.18%），纳入患者以二线治疗的患者为主（21/34, 61.76%），且二线治疗组的患者大多接受ICI单药治疗（20/21, 95.24%）。本研究仅纳入少量以ICI作为一线治疗的患者（8/34, 23.53%），且大部分患者接受ICI联合化疗（6/8, 75.00%）。

已发表的针对二线的NSCLC患者的临床试验数据显示，不同的ICI单药二线治疗晚期NSCLC的中位PFS波动在2.3个月-3.5个月^[7-9]^；而在KEYNOTE-010中^[10]^，依据PD-L1的表达状态，不同剂量帕姆单抗治疗组（2 mg/kg和10 mg/kg）在PD-L1表达大于1%的NSCLC患者中的中位PFS分别为3.9个月和4.0个月，在PD-L1表达≥50%患者中的中位PFS分别为5.0个月和5.2个月，提示在二线治疗中PD-L1高表达患者较低表达者的PFS有延长。

本研究中以ICI作为二线治疗的人群，中位PFS为5.87个月，中位PFS较前所述的临床试验均明显延长。考虑原因可能与PD-L1表达相关，本研究二线免疫治疗组中PD-L1%表达≥50%的比例为28.57%（6/21），而在CHECKMATE-017^[11]^与CHECKMATE-057^[7]^中PD-L1%表达≥50%的比例分别为12.59%与22.60%。多个临床试验结果分析表明PD-L1表达情况与ICI治疗反应和预后相关，PD-L1是目前NCCN指南推荐的一线免疫治疗疗效相关的标志物。对于晚期NSCLC患者二线免疫治疗，目前指南尚不推荐PD-L1检测作为指导治疗的依据。但本研究的分析显示在二线免疫治疗组中PD-L1%表达≥50%的患者其中位PFS优于PD-L1%表达 < 50%的患者（7.98个月*vs* 4.91个月，*P*=0.268），尽管统计学无差异。本研究及KEYNOTE-010均提示在二线治疗中，PD-L1高表达患者可能更能从ICI治疗中获益。因此，在实际临床应用中，即使是二线患者，明确PD-L1表达状态可以帮助筛选可能从ICI治疗获益的优势人群。

本研究二线免疫治疗组患者中位PFS的显著延长还可能与纳入的驱动基因阳性患者的比例相关，本研究中二线治疗组未纳入*EGFR*突变或*ALK*重排阳性的患者。但在CHECKMATE-057试验中^[7]^，其纳入的驱动基因阳性患者的比例为29.11%，而KEYNOTE-010试验中^[10]^，哌姆单抗治疗组（2 mg/kg, 10 mg/kg）驱动基因阳性患者的比例分别为8%和9%，而在其PD-L1表达≥50%亚组人群中比例分别为6%和9%。且这两个临床试验的亚组分析显示，在EGFR突变阳性人群中单药ICI相比于多西他赛，其总生存期（overall survival, OS）无显著提升。驱动基因变异与免疫治疗之间的关系复杂，仍有许多尚待回答的问题。一项回顾性研究^[12]^发现，*EGFR*突变或*ALK*重排阳性的NSCLC患者使用ICI单药后的ORR仅为3.6%，与此相对，在EGFR及ALK阴性或未知的患者中，其ORR为23.3%。另一项回顾性研究^[13]^发现*EGFR*突变或*ALK*重排阳性的NSCLC患者从ICI单药治疗中获益有限，而*KRAS*突变患者可以从ICI单药治疗中获益。同样的，一项纳入3, 025例晚期NSCLC患者*Meta*分析显示，相比于多西他赛，单药ICI不能提升*EGFR*突变阳性患者的OS，但可提升*KRAS*突变阳性患者的OS^[14]^，有研究^[15]^认为这种差异与不同突变基因对肿瘤微环境的影响相关，特别是对于肿瘤浸润淋巴细胞活性的影响有关。而且有研究^[16]^发现，在*EGFR*突变阳性患者中PD-L1表达情况与ICI治疗获益无关。在一线治疗方面，一项旨在评估ICI单药在PD-L1%表达≥1%且*EGFR*突变阳性晚期NSCLC患者一线治疗效用的Ⅱ期临床试验因疗效不佳（0/10）而提前终止^[17]^。总的来说，在常规临床实践中，排除*EGFR*突变/*ALK*重排的患者，ICI单药治疗的应答率应该会更高。本研究仅纳入1例*EGFR*基因21外显子L858R突变的患者，在一线靶向治疗进展后，查血无T790M突变，接受6个周期二线化疗后进展，三线治疗接受了5个周期纳武单抗治疗和腰椎转移灶手术切除及放疗。此患者PD-L1表达情况为0%，三线免疫治疗的PFS达到5.1个月。IMPOWER150试验^[5]^的亚组分析中，有80例接受过EGFR-TKI治疗病失败的EGFR突变阳性的患者，接受免疫治疗联合双药含铂方案+贝伐珠单抗治疗的中位PFS达10.2个月。这提示ICI联合其他治疗如放疗、化疗或抗血管治疗或许能使驱动基因突变阳性的晚期NSCLC患者获益。

本研究结果显示TNM分期以及远处转移部位与PFS有关，这与既往的回顾性研究一致。一项日本的回顾性研究^[18]^显示接受ICI治疗的晚期NSCLC患者其肝转移和肺转移的存在与较差的PFS相关。而最近韩国的一项回顾性研究^[19]^更进一步确认了在接受ICI治疗的晚期NSCLC患者中，肝转移和脑转移的存在与较差的OS相关。本研究数据显示发生胸膜转移与较差的PFS相关，甚至较肺外转移更差，因本研究为回顾性分析，样本量偏小，需更大规模的研究或成熟的OS数据来进一步证实。

一些研究^[19, 20]^结果表明irAE的发生与更好的预后相关，但本研究未能得出同样的结果。本研究中还将皮肤和内分泌相关的irAE行统计学分析，发现出现皮肤相关irAE患者的PFS较未发生者明显延长（7.86个月*vs* 4.93个月，*P*=0.056），但无统计学差异。本研究显示免疫相关性肺炎的发生率为11.76%（4/34），高于临床试验中报道的发生率3%-5%。而最近一项回顾性研究^[21]^也同样发现ICI应用于晚期NSCLC治疗时其免疫相关肺炎的发生率达到19%，远高于临床试验的报道。在ICI实际应用中临床医生应警惕免疫相关性肺炎的发生。另外，本研究中有3例发生致死性irSAE，其中2例发生了免疫相关性肺炎，1例出现了免疫相关性心肌炎。发生致死性免疫相关肺炎的2例患者分别接受纳武单抗和阿特朱单抗治疗，2例均以ICI作为三线及以上治疗方案，在接受免疫治疗前行多线化疗。2例患者均在接受ICI治疗5个周期后出现喘憋等症状，行类固醇静滴和/或口服后，症状及肺部病变影像学表现较前好转，但最终由于继发严重肺部感染而导致死亡。在一项针对PD-1/PD-L1抑制剂引起肺炎的回顾性研究中，12例患者出现≥3级免疫相关肺炎，同样予以停用ICI和类固醇激素治疗，7例患者病情好转，5例患者在肺炎治疗过程中死亡，其中1例患者死于肿瘤进展，1例患者死于难治性肺炎，3例患者死于与免疫抑制相关的感染^[22]^。这提示在临床中使用类固醇治疗irAE时，应注意机体的免疫抑制状态，警惕继发感染。发生致死性免疫相关心肌炎的1例患者接受了ICI为二线治疗，在2个周期的纳武单抗单药治疗后出现发热、全身酸痛等症状，心电图提示“急性心梗”，后经冠脉造影、心电图、心肌酶等检查排除冠心病、急性心肌梗死，考虑免疫相关心肌炎，后患者因大面积心肌损伤、横纹肌溶解、急性肝肾损伤等死亡。免疫相关心肌炎常发生于免疫治疗的早期，一旦发生便具有高死亡风险，一篇纳入101例发生免疫相关心肌炎患者的文章^[23]^分析表明其致死率为46%，且多个ICI联合治疗的致死率高于ICI单药治疗（67% *vs* 36%）。临床中及时识别和处理严重的irAE是非常重要的。目前，针对ICI引发的irAE的研究在进行中，但缺乏预测患者出现严重irAE的有效预测因子，期待未来的研究可进一步阐明irAE发生的机制和特点，筛选出ICI治疗获益的优势患者，实现ICI的精准治疗。

本研究是对临床实践中晚期NSCLC患者应用PD-1/PD-L1抑制剂的一项回顾性研究，受限于药物的可及性和适应证等因素影响，存在以下局限性，回顾性单中心研究、样本量偏小、治疗药物种类多、OS数据不成熟等。该研究虽有缺陷但其提供了临床中实际应用ICI患者的真实数据和经验，临床医生在临床应用ICI时需更加关注不良反应，尤其需警惕致死性严重不良反应的发生；临床医生为患者制定免疫治疗方案时需充分评估患者，与患者家属沟通风险与获益的可能，让免疫治疗改变更多晚期肺癌患者的生活。
